# Phospholipase A2—A Significant Bio-Active Molecule in Honeybee (*Apis mellifera* L.) Venom

**DOI:** 10.3390/molecules30122623

**Published:** 2025-06-17

**Authors:** Mara Muntean, Adrian Florea

**Affiliations:** Department of Cell and Molecular Biology, Faculty of Medicine, “Iuliu Hațieganu” University of Medicine and Pharmacy, 6. Louis Pasteur St., 400349 Cluj-Napoca, Romania; muntean.mara@elearn.umfcluj.ro

**Keywords:** phospholipase A_2_, honeybee venom, *Apis mellifera*, cell membrane, phospholipids, allergen, toxicity, anti-PLA_2_ molecules

## Abstract

Phospholipase A_2_ (PLA_2_) is a prevalent molecule in the honeybee venom. Its importance is reflected by the number of scientists focused on studying it from various points of view. This review summarises a significant amount of data concerning this fascinating substance. Firstly, the origin and occurrence of PLA_2_, with similarities and differences among species or populations of bees are highlighted. Next, its synthesis, post-translational processing and structural features are described, followed by the PLA_2_ availability. In a larger section, the multiple effects of honeybee venom PLA_2_ are detailed, starting with the main ability as an enzyme to interact with biological membranes and to hydrolyse the sn-2 ester bond in 1,2-diacyl-sn-3-phosphoglycerides; the docking process, the substrate binding and the catalytic steps are analysed too. Then, the pro-/anti-inflammatory effect and allergenic property, the anticoagulant effect and the involvement of PLA_2_ in apoptosis are revised. Selected antiviral, antibiotic and antitumoral effects of PLA_2_, as well as its use in immunotherapy are mentioned as beneficial applications. Additionally, the mechanisms of toxicity of PLA_2_ are presented in detail. Finally, a number of anti-PLA_2_ compounds are enumerated. In each section, the features of the honeybee venom molecule are discussed in relation to PLA_2_s from other species.

## 1. Introduction

Venoms are complex mixtures of many inorganic and organic molecules, produced and used by numerous animals, very distant on the evolutionary scale. The venom is used either as a defensive weapon—to fight against predators, or for attack—to immobilise and digest prey [1]. Bees use their venom mostly to defend the hive, and therefore they developed a complicated sting apparatus and a particular strategy, based on high coordination [2]. Also, their venom was adapted to this purpose. Its chemical composition and the amount released during stinging, along with a relatively reduced level of aggressivity (except for the Africanized bees—which resulted after human intervention) contribute to discouraging their mammalian enemies, rather than killing them [3,4].

Among the multiple substances present in the dry (crystallised) honeybee (*Apis mellifera* L.) venom (HBV), phospholipase A_2_ (PLA_2_) stands as the second one in terms of concentration and roles, after a peptide named melittin. Neumann et al. [5] characterised the HBV for the first time and, a few years later, Habermann and Neumann [6] reported the presence of PLA_2_ in the HBV. Then, after Habermann published his famous paper in Science [7], the interest of scientists for this valuable natural compound increased exponentially, and the 1990s represented the golden decade in the study of HBV, mainly of HBV PLA_2_ (hvPLA_2_). The methods to study the HBV and its components evolved from filter paper electrophoresis, diversified and culminated with high-performance liquid chromatography (HPLC) and cloning of the hvPLA_2_ in bacteria [8]. In the meantime, the methods used for studying the effects of HBV or hvPLA_2_ alone advanced from blood testing to the production of monoclonal antibodies, electrophysiology and transmission electron microscopy. Even though the number of publications on this topic decreased during the last 10–15 years, the subject still remains of actual scientific interest, due to the new medical relevance of the hvPLA_2_ [9,10,11,12].

In our laboratory, there is a long-standing interest (originating from the above-mentioned golden decade) in the study of histological, histochemical, ultrastructural and physiological effects of HBV in different organs or tissues, in experimental conditions. All this activity was concretised in the publication of 16 papers in Romanian journals not indexed in WOS (between 1996 and 2005), and later in other 6 papers in WOS-indexed journals. Among the formers, some reported interesting effects of hvPLA_2_ in vivo [13,14] and in vitro [15]. As a logical consequence, an important amount of scientific literature regarding hvPLA_2_ has been accumulated, from different databases and using various keywords, that represents the foundation of the present work. We analyse here the hvPLA_2_ structure and its many effects in relation to other similar molecules belonging to the same family, but present in other species. Finally, a number of anti-PLA_2_ molecules are discussed.

## 2. PLA_2_—A Common Molecule in Different Organisms

PLA_2_, also called phosphatidilcoline-2-acyl hydrolase, or lecithinase A, is an enzyme commonly found in living beings. Historically, it was first isolated from the pancreas and described by Bókay in 1878 [16]. A few decades later, PLA_2_ was also identified in cobra venom, and, in the following years, in many invertebrate and vertebrate species [17,18,19]. Despite their very different origins, PLA_2_s display similar structures, and, more importantly, they share a common function: the capacity to catalyse the hydrolysis of the sn-2 ester bond in 1,2-diacyl-sn-3-phosphoglycerides (PLA_2_ EC 3.1.1.4. [20]), thus generating free fatty acids and lysophospholipids. Apart from PLA_2_, other two enzymes with similar activities exist: PLB (that hydrolyses phospholipids at either the sn-1 or sn-2 positions) and PLC (that removes the phosphate group from phospholipids) [21].

PLA_2_s have been categorised into 11 classes and 23 subclasses, based on several criteria, such as their source, structural features and effects [18], and later into 15 groups. They cluster in five main categories or types: secreted PLA_2_s (sPLA_2_s), cytosolic, Ca^2+^-independent, platelet-activating factor acetyl hydrolase, and lysosomal PLA_2_s (for an outstanding review of PLA_2_ classification and tools used for this, see [22]).

So far, more than 150 different PLA_2_s have been characterised in the venoms of different species [23]. Venom PLA_2_s are all Ca^2+^-dependent sPLA_2_s, relatively small molecules ranging between 13 and 19 kDa. sPLA_2_s comprise 17 known distinct isoenzymes [24,25,26]. The hvPLA_2_ is a sPLA_2_ that belongs to class IIIA [18,27,28,29,30].

## 3. PLA_2_ Is a Major Molecule of HBV

### 3.1. Bees and Their Venoms

Michener [31] classified bees (insects from the order *Hymenoptera*) in 17,533 species from around the world. They were grouped into 443 genera differentiated into diverse lineages, with or without venom. The most relevant are the 11 venom-producing species of genus *Apis* (family *Apidae*), including *A. mellifera* (the European honeybee, with 33 subspecies), *A. cerana* (the Asian honeybee), *A. florea* (dwarf honeybee), and *A. dorsata* (giant honeybee) [31,32].

The venom is used by worker bees to defend themselves and their colony, while queens only use it during fights with other queens [33]. Due to its beneficial properties, the bee venom was considered for centuries—particularly in Eastern Europe and Asia—as a traditional drug in the treatment of certain arthritic ailments [34,35,36]. In trying to explain the venom action on the human or animal body, several prominent scientists have studied its composition. The venom secreted by bees is a highly concentrated mixture of at least 18 organic and inorganic substances including proteins (enzymes), peptides, amino acids, sugars, lipids and acids [5,7,37,38,39,40,41], most of them with biochemical or pharmacological activity [7,42,43].

### 3.2. hvPLA_2_—Differences

hvPLA_2_ has been identified as an important molecule accounting for 11–15% of the dry venom of worker bees [6,7]. Like the other components of HBV, hvPLA_2_ is synthesised by cylindrical secretory cells in the bee venom’s main gland. This gland is thin, convoluted, and bifurcated, located in the abdomen between the rectum and the ovaries, and attached to the sting apparatus [37,44,45]. After synthesis, HBV is collected and stored in a reservoir of this gland. According to Owen and Bridges [46], the secretory cells undergo ultrastructural changes in adult bees, a process that is preceded by a decrease of 50% in the protein content of the venom glands; these data were further confirmed by Abreu et al. [47]. Additionally, studies by Owen et al. [48], and Li et al. [49] showed that PLA_2_ is synthesised in *A. mellifera* mainly after hatching, and within 10 days it reaches its maximal level in the venom reservoir of worker bees (about 40 μg); this level is then maintained throughout the rest of their life. Li et al. [49] also reported undetectable PLA_2_ mRNA during the later adult stage of *A. cerana*, suggesting either an arrest in PLA_2_ synthesis, or a gland involution. The synthesis and storage of PLA_2_ in the venom sac in *A. cerana* follow the same steps as in *A. mellifera*, with maximal values found in the 10th day. However, the enzyme was detected in a lower amount in comparison to *A. mellifera*—only 10–12 μg [49]. These data were consistent with the differences in the total venom content of the sac of bees from the two species: 138 μg [49], or 147 μg [50] in *A. mellifera*, and 43 μg in *A. cerana* [49], respectively. Furthermore, Palma and Brochetto-Braga [51] observed both qualitative and quantitative biochemical differences between the venom of 3 races of *A. mellifera*: *A. m. ligustica*, *A. m. adansonii*, and *A. m. scutellata* (Africanized bees). Apart from this, Schumacher et al. [52] reported a high variability in venom quantity among individual mature bees, and across different colonies, with an average of 134.1 μg (range 38–330 μg) in *A. m. mellifera* and 98.2 μg (range 34–200 μg) in *A. m. scutellata*. The PLA_2_ content also showed the same variability: 11.1% (1.8–27.4%) from dry weight in *A. m. mellifera*, and 18.1% (range 5.1–31%) in *A. m. scutellata*. Additionally, small seasonal differences in PLA_2_ concentration were observed in *A. m. mellifera* [53] and in *A. m. scutellata* [54], along with variations based on the age of the bee [48,55].

These differences are mainly thought to be due to the involvement of *A. mellifera* in the defence of their bigger colonies (against large mammals and other vertebrates) as compared to *A. cerana* and to the higher fighting skills of the European bees [49]. On the other hand, in the Africanized bees (recognised as the most aggressive), multiple stinging, rather than increased venom amount, potency or delivery seems to be the cause of the serious reactions following their attacks [50].

Unlike in workers, it was found that queen venom contains much less PLA_2_ [56]. Thus, it was concluded that different control mechanisms influence the production of PLA_2_ in these castes of identical genotype. The genes encoding the PLA_2_ from the venom of different species of bees have been sequenced and cloned (*A. mellifera* and *A. cerana* by Shen et al. [57], *A. cerana* by Li et al. [49] that also reported differences from *A. mellifera*). Moreover, the genes encoding PLA_2_ from many other species of animals (with or without venoms) have been studied so far [58,59,60].

### 3.3. hvPLA_2_—Synthesis and Proteolysis

In *A. mellifera*, hvPLA_2_, encoded by a single-copy gene/haploid genome, is synthesised in the rough endoplasmic reticulum as a bigger precursor of 167 amino acids, with a molecular weight of 19.058 kDa [61]:

MQVVLGSLFL^10^ LLLSTSHGWQ^20^ IRDRIGDNEL^30^ EERIIYPGTL^40^ WCGHGNKSSG^50^ PNELGRFKHT^60^ DACCRTHDMC^70^ PDVMSAGESK^80^ HGLTNTASHT^90^ RLSCDCDDKF^100^ YDCLKNSADT^110^ ISSYFVGKMY^120^ FNLIDTKCYK^130^ LEHPVTGCGE^140^ RTEGRCLHYT^150^ VDKSKPKVYQ^160^ WFDLRKY^167^

**Figure 1:**
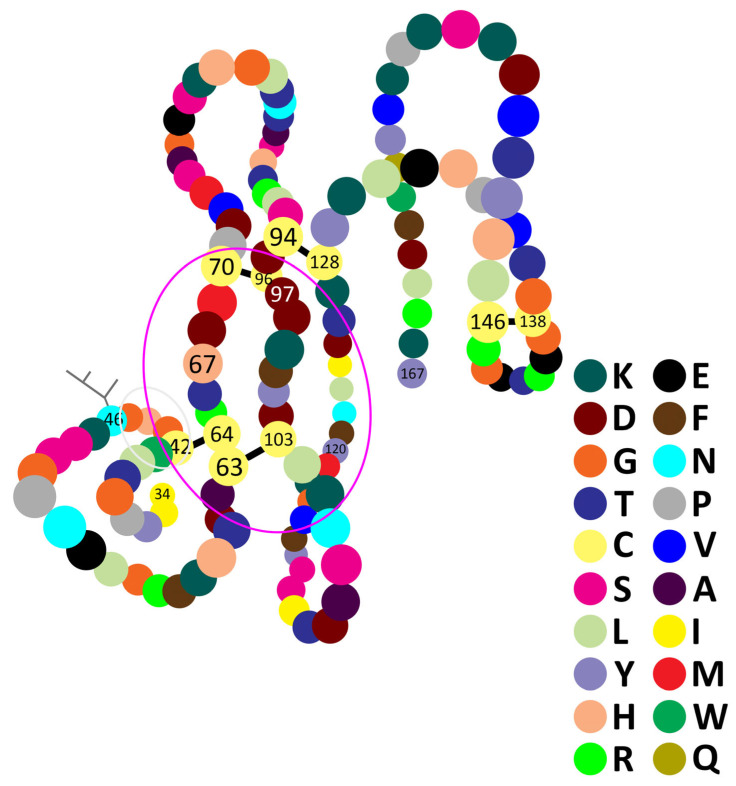
Primary, secondary and tertiary structures of the honeybee venom phospholipase A_2_ (hvPLA_2_) imagined based on the numerous structures available in [61]. See the text for the significance of numbers. Black lines, disulfide bonds; branched lines, glycosylation motif; grey circle, Ca^2+^-binding domain; pink circle, possible area of the catalytic site.

This precursor is next processed to the mature form by proteolytic cleavage: the signal peptide of 18 amino acids (1–18), and then a smaller propeptide of 15 amino acids (19–33) are removed; thus, the secreted protein has 134 amino acids (numbered in Figure 1 from 34 to 167) and a molecular weight of 15.249 kDa [61]. Also, in [61] the lengths of the signal and of the mature protein are available.

Additional experimental evidence showed 31 sites of proteolysis in the structure of the hvPLA_2_. But, while Habermann [7], King [62] and Annand et al. [63] found 14 chymotryptic and 17 tryptic attack points, Shipolini et al. reported 13 and 18, respectively [64].

These data were obtained in two different ways. One method, used by Kuchler et al. [65] was based on analysis of the cDNA for PLA_2_ from BV glands. The other method involved studying the purified secreted hvPLA_2_: Scott et al. [66] and Shipolini et al. [64] reported a molecule of 128 amino acids, while studies of Habermann [7] and Annand et al. [63] showed 129 amino acids forming the polypeptide chain. Apart from two N-D replacements, the amino acid sequence determined by Kuchler et al. [65] is identical to the one previously reported by Shipolini et al. [64] in the N- and C-terminal regions, while several differences have been recorded in the central part of the molecule.

All these small discrepancies could stem from the different resolutions of the methods employed by the researchers.

The primary structure of hvPLA_2_ was found to be similar in a ratio of 31% to the group III of human cellular sPLA_2_s [67,68,69]. On the other hand, in its N-terminal region 60% of the amino acid sequence is common with those of PLA_2_s from the venoms of the *Rhopilema nomadica* medusa and of the *Heloderma* lizard [70]. While sPLA_2_s in groups I and II are highly homologous [30], hvPLA_2_ shares little similarity with them, except for a few regions—the catalytic site, the Ca^2+^ binding loop, and certain cysteine residues [65]. Kuchler et al. [65] also discussed other aspects concerning the homology of different domains of the hvPLA_2_ with their counterparts in vertebrate PLA_2_s and concluded that the common features are typical for proteins having a common evolutionary origin.

### 3.4. hvPLA_2_—Glycosylation

hvPLA_2_ is a glycoprotein resulting from the co-translational N^46^-glycosylation (see Figure 1). The sugar motif consists of mannose, N-acetylglucosamine, fucose (that is alpha 1–6 and/or alpha 1–3 linked to the N-acetylglucosamine), galactose and a low amount of N-acetylgalactosamine (accounting for about 10% of the PLA_2_ oligosaccharides) [62,64,66,71,72,73,74].

According to Li et al. [49], the glycosylation pattern in *A. mellifera* results in the formation of four types of PLA_2_s, with different molecular weights, but Altmann et al. [75] characterised three hvPLA_2_ isoforms in this species, of about 16, 18 and 20 kDa. The differences between them were mainly given by their glycosylation patterns: the isoform with the lowest molecular weight is not glycosylated in N^13^ as the other two are, while the heaviest contains N-acetylgalactosamine. Shipolini et al. calculated a molecular weight of 15.800 kDa for the glycosylated PLA_2_ in *A. m. mellifera* [64]. The controversial data could result from the accuracy of the working conditions as well as from the different resolutions of the methods employed. Comparatively, the glycoprotein has 15.249 kDa in *A. m. carnica* [76]. In *A. cerana*, the glycosylation leads to the occurrence of an enzyme with a higher molecular size, often correlated with ageing. However, this glycosylation process was found to be different between the two species [49].

### 3.5. hvPLA_2_—Folding

Li et al. [49] showed complex patterns of post-translational modifications of PLA_2_, identical among the individuals of *A. mellifera*. In *A. cerana*, nevertheless, the post-translational modifications were found to differ within the same colony, between different colonies and in comparison to *A. mellifera*.

The N-terminal segment of hvPLA_2_ arrives, after the three-dimensional folding, at the central part of the enzyme, which represents the catalytic centre, also containing the Ca^2+^ binding domain with a conserved XCGXG motif [63,77] (see Figure 1 amino acids 41–45). Because both consist of hydrophobic amino acids, they participate in the formation of a hydrophobic channel at this level [78].

Ten cysteine residues contribute to the particular folding that generates the secondary and tertiary structure of PLA_2_. They form 5 disulfide bonds in positions: 42–64, 63–103, 70–96, 94–128, and 138–146 [63,65,66,79] (as depicted in Figure 1). This leads to one of the main differences from other classes of PLA_2_ that contain seven disulfide bonds with another pattern [63,80]. Furthermore, the sizes and shapes of the amino acids also lead to the formation of three α-helix regions: K^58^-H^67^; C^94^-N^106^; I^111^-N^122^ (see Figure 1 and [61]). On the contrary, hvPLA_2_ has many similarities with snake PLA_2_s (for review, see Montecucco et al., 2008 [81]). The crystal structure of hvPLA_2_ has also been well documented by comparison with the bovine pancreatic PLA_2_ [66], or with the enzyme from the Chinese cobra (*Naja naja atra*) [82]. For an instructive 3D representation of the hvPLA_2_, see [61]. X-ray crystallography has revealed a similar catalytic domain with other classes of PLAs [66].

### 3.6. Availability

**Figure 2:**
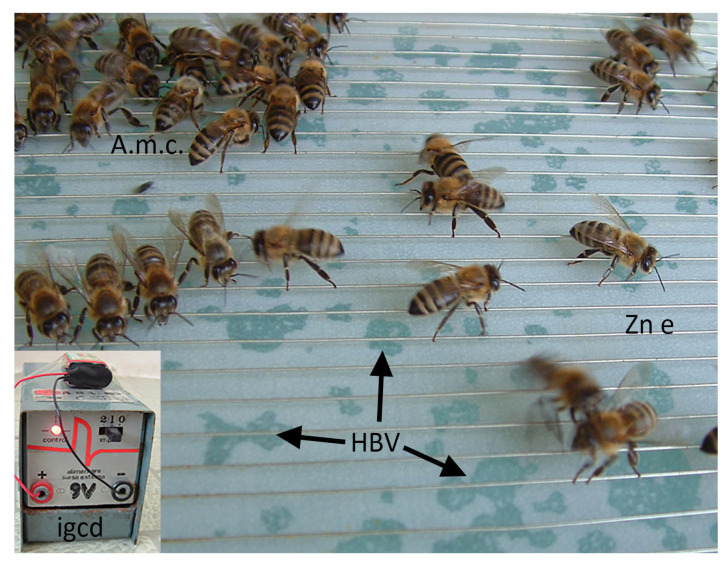
Venom collection device using the electric stimulation of bees. A.m.c., *Apis mellifera carpatica*; HBV, honeybee venom under a latex membrane; igcd, impulse generator of the collecting device; Zn e, zinc electrodes. (personal archive A.F.).

Even though the hvPLA_2_ is secreted by bees in low amounts, their extremely high number allows the collection of reasonable quantities of venom, using, as a main standardised method, the electric stimulation of the stinging reflex of bees [47,83], as represented in Figure 2. Recent data indicated that the amount of venom correlated with the position of the bee venom collector frame [84], as well as with the temperature [85]. Scaccabarozzi also reported differences in HBV weight and protein diversity caused by both temperature and other ecological factors [86]. From the dry HBV, the enzyme can be further purified with high yield through preparative HPLC [87]. An alternative way of directly obtaining the hvPLA_2_ is by bioengineering based on cloning the bee PLA_2_ gene in the bacteria *E. coli* [8]. The commercially available lyophilised powder containing hvPLA_2_ is useful for accurate experimental activities since it is very stable at −20 °C [87].

The aqueous formulation could also be used, but the enzyme stability is lower at the same temperature [88]. The catalytic activity of the hvPLA_2_ is documented: “One unit will hydrolyze 1.0 μmol of soybean L-α-phosphatidylcholine to L-α-lysophosphatidylcholine and a fatty acid per min at pH 8.9 at 25 °C” [87]. On the other hand, Nair et al. studied the thermal stability of hvPLA_2_ and noted a maximum activity at 65 °C using egg yolk as substrate and at 60 °C in the presence of dicaproyl and dipalmitoyl lecithin [89].

In our previous studies, we tested the lyophilised hvPLA_2_ (product number P9279, m.w. 14,500 Da, purity 1956.57 units/mg protein) from Sigma-Aldrich (St. Louis, MO, USA). The obtained results published so far (as well as others in analysis) were impressive, helping our progress in understanding the effects of the HBV on the mammalian organism.

## 4. Effects of hvPLA_2_

**Figure 3:**
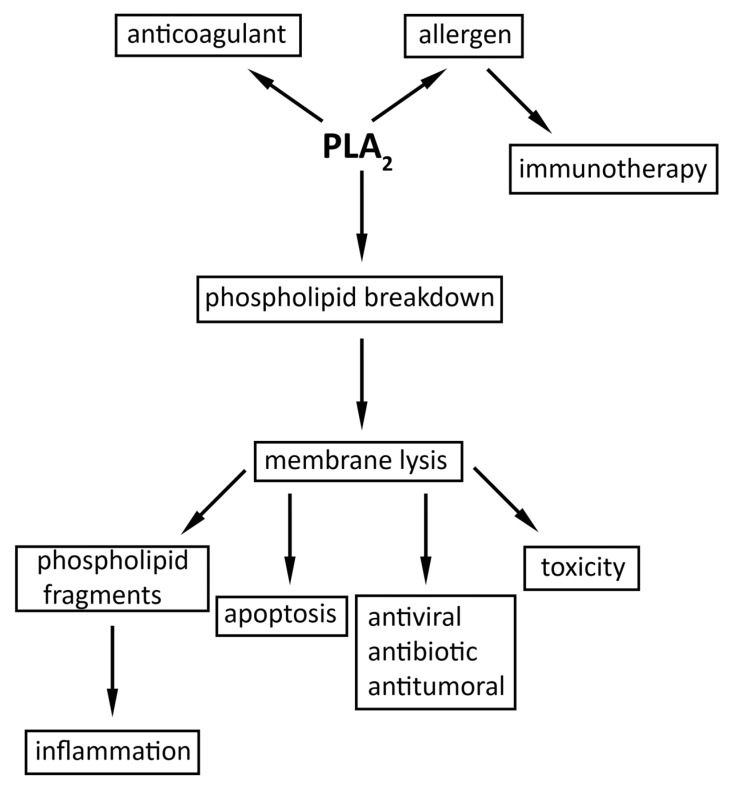
Main effects of the hvPLA_2_.

In consistency with its occurrence in so many different classes of organisms so distant phylogenetically, the functions of PLA_2_s are very diverse. The most studied PLA_2_s are those from venomous insects, playing various toxic roles, and those from mammals. For the latter ones, it was noted their involvement in a range of cellular processes, from lipid digestion [90] to inflammation [91,92], or control of cellular proliferation [93]. Since the purpose of this review is to deal mainly with hvPLA_2_, we hereby discuss its functions (Figure 3), but in relation to other PLA_2_s.

### 4.1. Breakdown of Membranes

One of the first noted effects of the HBV was its ability to induce haemolysis and cell membrane permeability [94,95,96]. This feature is conferred by PLA_2_, as well as by other substances, especially by melittin—a peptide which is the main component of bee venom. hvPLA_2_ has harmful effects on the complex lipids in animal or human organisms [7,41].

Thus, its final effect is damaging the cell or its organelles. It has low activity in the presence of phospholipid monomers, but it is activated by high concentrations of substrate molecules—micelles, cell membranes, vesicles with phospholipid bilayers, etc. [19]. In cells, the sn-2 position of phospholipids often contains polyunsaturated fatty acids that, when released by the PLA_2_-catalysed hydrolysis, can be further metabolised into eicosanoids and other bioactive lipid mediators [97]. Additionally, the resulting lysophospholipids may play various significant biological roles [98].

### 4.2. Interaction with Membranes

The interaction of PLA_2_ with membranes has been studied on enzymes from various organisms, and the comparison of hvPLA_2_ with other phospholipase structures provides compelling evidence for a common catalytic mechanism [66,82,99,100].

hvPLA_2_ is a basic, water-soluble protein, that must attach itself to membranes in order to interact with phospholipids and to hydrolyse them. Its solubility in water and its activation by phospholipids in high concentrations have suggested a mechanism of enzymatic catalysis occurring in two phases. The first step is the interfacial binding of the enzyme, followed, in the next step, by the catalytic reaction. Therefore, from a functional point of view, PLA_2_ displays two distinct, very important domains. The first one, responsible for substrate recognition, surrounds the second one, the catalytic site; both are oriented in the same direction [28,101].

**Figure 4:**
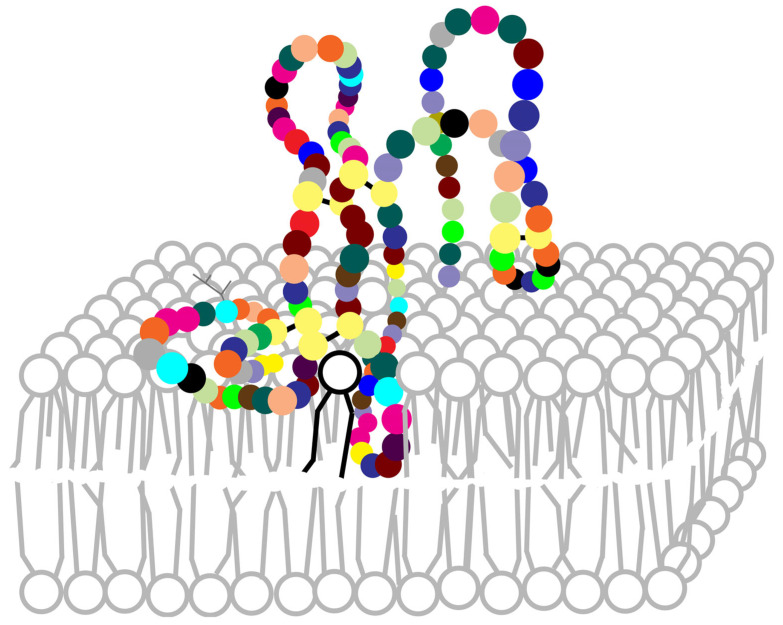
Interaction of the hvPLA_2_ with a biological membrane.

The contact of PLA_2_ with membranes triggers specific modifications in the three-dimensional structure of the enzyme, particularly in the N-terminal region. While this region of the molecule is randomly unfolded in water, it turns into an α-helix structure when binding to the phospholipid surface [19,28]. Based on studies of enzymatic kinetics performed using an electron paramagnetic resonance spectroscopy technique, Lin et al. [101] established that PLA_2_ attaches to the water-lipid interface, without integrating into membranes. By measuring the lateral pressure of the phospholipids in the monolayer exposed to PLA_2_, they found minor modifications after interfacial binding, with values comparable to those of the intact layer. These data suggest only a reduced infiltration of PLA_2_ in the outer membrane monolayer. One year later, Ball et al. [102], followed by Ahmed et al. [103] developed a model for hvPLA_2_ docking on membranes. This model postulates that the acyl residue behaves like a hydrophobic anchor: it penetrates the lipid surface and partially inserts itself into the bilayer (as represented in Figure 4). Thus, through this substrate-binding process, the lytic power of PLA_2_ is significantly increased. Notably, this first association of PLA_2_s with membranes is mainly based on hydrophobic affinities and interactions, and not on electrostatic ones [19,28,101,104,105]. Jackman et al. [106] suggested that the initial hvPLA_2_ docking can be followed by a “lipid desorption”, process determined by electrostatic interactions, thus leading to membrane lysis. Additionally, Maggio [107] showed that external electrostatic fields, as well as the charges of the lipid molecules of the membrane, can influence the enzyme’s activity. Experiments with amino acid replacements in the structure of hvPLA_2_ proved that changes in the charge of the residues at the membrane-binding site can also affect how strongly the enzyme interacts with the substrate [108], the mutant enzyme with anionic amino acids in the binding domain attaching more tightly. Furthermore, the actual catalytic activity seems to be enhanced by the presence of a long-chain fatty acid in the binding site consecutive to conformational changes in the protein and not due to hydrophobic interactions with the substrate [109]. The enzyme is next involved, in the presence of 2 Ca^2+^ ions [78,82,94], in multiple interactions with phospholipids, during which the hydrophobic channel expands, and the interfacial binding domain is remodelled.

Y^57^ of the human sPLA_2_ plays an important role in this process [78]. Ball et al. [102] confirmed that the Ca^2+^-binding region, which is rich in hydrophobic residues [110], also contributes to the process of enzyme docking to the membrane. The remaining chain lines up parallel to the lipid bilayer, in the proximity of the polar headgroups of the phospholipids. Even though Ca^2+^ ions are not necessary in the first step of PLA_2_ docking, they represent an important cofactor required for the attachment of phospholipid molecules to the catalytic domain of the enzyme, as well as for the initiation of the enzymatic reaction [28,105]. The results of Scott et al. [66] indicated that cobra venom PLA_2_ facilitates “substrate diffusion from the interfacial binding surface to the catalytic site rather than an allosteric change in the enzyme’s structure”. For the PLA_2_ from another snake (*Agkistrodon contortrix laticinctus*), it was shown by Ambrosio et al. [111] that a fatty acid adsorbed to the active site triggers conformational changes in the C-terminal region, responsible for the Ca^2+^-independent activity of the enzyme. Data reported by Nabemoto et al. [112] suggest that sPLA_2_s are inactive in the first stages of interaction with membranes and their activation is triggered by a modification of their disulfide bonds.

Studies performed on different types of liposomes have revealed further aspects of PLA_2_ binding to membrane surfaces. Thus, Ghomashchi et al. [104] reported that hvPLA_2_ binds preferentially to anionic vesicles versus phosphatidylcholine vesicles. Holopainen et al. [113] have shown that the lytic effect of PLA_2_ is extremely powerful in the case of unilamellar liposomes, where a pronounced tendency of aggregation of the neighbouring vesicles was also observed. The multilamellar liposomes displayed only minor variations in size, thus suggesting that the hydrolytic effect of the enzyme only concerns the outer monolayer. Furthermore, it was observed that PLA_2_ is temperature-dependant: its lytic activity is significantly elevated in liposomes in liquid crystal state, and irrelevant in liposomes in gel state. Another modulating factor is the lateral equilibrium pressure of lipid molecules in membranes. This remark can be supported by the lack of modifications in phospholipid monolayers at surface pressures of over 30 mN/m; in the gel state, the lateral equilibrium pressure exceeds the capacity of the enzyme to insert into lipid layers [113]. Billy et al. [114] obtained similar results working on washed platelets.

### 4.3. Mechanism of Phospholipids Hydrolysis

The catalysing mechanism of hvPLA_2_ has been well documented [66,78]. X-ray crystallography data revealed a similar catalytic centre with other classes of PLAs, proving their common action mechanism [66]. Thunnissen et al. [115] described the changes in the crystalline three-dimensional structure of PLA_2_ during the process of substrate binding to the catalytic domain. After docking on the membrane, the catalytic site of the hvPLA_2_ surrounds the hydrophilic group of a single phospholipid molecule via specific interactions. Ambrosio et al. [111] have shown that the position initially occupied by the sulphate ions in a modified hvPLA_2_ corresponds to that where the phosphate group of the phospholipid temporarily attaches. They also reported significant conformational changes in the Y^52^, K^53^, and H^68^ residues involved in this process for the serpent *A. contortrix laticinctus* (broad-banded copperhead) PLA_2_.

R and K residues (in a total number of 18 in hvPLA_2_ and 23 in the human sPLA_2_ from certain cells during inflammation) play important roles in the interaction with lipid membranes and in the formation of protein-lipid complexes. Replacement through reversible mutations of the cationic residues R^7^ and K^10^ from the interfacial binding area of the human cellular sPLA_2_ with E was followed only by a moderate decrease of its capacity to bind the lipidic substrate from phospholipid anionic vesicles. These data, also confirmed by certain experimental results of enzyme binding to micelles (phospholipid-detergent), support the finding that a higher number of R and K residues are involved in this kind of protein-lipid assembling [116].

The active catalytic site in the hvPLA_2_ molecule is located in its central region [66,67,117]. Annand et al. [63] showed the paramount importance of three amino acids in the active site of hvPLA_2_ (H^67^, D^97^ and Y^120^, see Figure 1). A key function is however performed by the H. Its capital role was proved using mutant, inactive enzymes, with H^67^ replaced by another amino acid. On the other hand, the enzymes without the two other essential amino acids (D^97^ and Y^120^) still maintained an efficient catalytic activity. Histidine probably also acts as a Brönsted base to deprotonate water, while D and Y establish hydrogen bonds with H^67^. On the other hand, Ghomashchi et al. [104] reported that the deletion of the large β-loop (residues 132–151) had no obvious consequences on interfacial binding and catalysis of hvPLA_2_.

This mechanism, described for the hvPLA_2_, was found to be similar for the sPLA_2_s in other species [110]. Thus, sPLA_2_s typically found in mammalian secretory fluids and snake venoms, have the active catalytic site made of H^48^ and D^99^ residues, as well as a D in position 49, which also binds the Ca^2+^ ions [82,118]. The replacement of the D^49^ by a lysine residue resulted in a catalytically inactive enzyme [119,120].

In their outstanding review, Berg et al. [28] summarised a high number of mutant variants of hvPLA_2_ as well as of sPLA_2_s from various species, emphasising their roles for a better understanding of the catalytic effect of the enzyme. Moreover, Schneider et al. [121] reported no differences in the catalytic activity of glycosylated (native, secreted form) and non-glycosylated (after enzymatic removal) hvPLA_2_.

The enzyme uses a H^+^ to trigger a nucleophilic attack on the sn-2 bond (β-ester bond) in the target phospholipid from the plasma membrane, leading to the release of unsaturated free fatty acids (arachidonic acid, among others) and lysophospholipids. The latters include lysophosphatidylcholine or lysolecithin—another “structural poison” with lytic action upon the neighbouring cells, maintaining an avalanche effect. As mentioned before, one unit of hvPLA_2_ produces one fatty acid each minute [7,19,38,82,117,122,123].

The enzyme displays a high affinity for many natural substrates, such as phosphatidylcholine, phosphatidylethanolamine, as well as for their plasmalogen analogues. By measuring the haemolytic power of the resulting lysolecithin, the quantitative estimation of hvPLA_2_ activity could be performed. However, this method is not an absolute one, because the intact remaining lecithin, as well as the cholesterol from membranes, are known as inhibitors of the haemolysis produced by lysolecithin [38].

The resulting metabolites (the fatty acid and the lysolecithin) behave as secondary messengers at low concentrations [124,125], while at high concentrations they are cytotoxic [124].

### 4.4. Pro-Inflammatory Effect

Another role of PLA_2_ is the production of inflammatory mediators [17,126,127]. The fatty acid and the lysolecithin resulting from the enzymatic activity of hvPLA_2_ can further serve as precursors for generating eicosanoids [126,128], platelet-activating factor, and lysophosphatidic acid, which in turn modulate many cellular processes [129]. Farooqui et al. [130] reviewed the multiple biological effects of all these molecules. Studies on mammalian sPLA_2_ revealed that it indirectly triggers the inflammatory process through the release of fatty acids. This, in turn, leads to increased vascular permeability, hemodynamic alterations and, further, organ injury [131]. Wery et al. [110] proved the presence of sPLA_2_ in various inflammatory exudates, in platelets and in the synovial fluid of patients suffering from rheumatoid arthritis. Furthermore, it induced tracheal inflammation and mucus production in an in vivo model of asthma [132]. Additionally, PLA_2_ was observed to also behave as an acute phase reactant, with clinical studies on post-surgical patients showing an immediate increase in the concentration of the enzyme, with a maximum in the second day post-operative [133].

### 4.5. PLA_2_—Allergen of Bee Venom

Many different populations (mainly in Europe, Asia and parts of Africa) have come in contact with bees and their venom since the dawn of humankind. Therefore, they learned how to avoid or to deal with them, and, at a certain moment in our distant history, how to take important advantages from apiculture. Apart from the intense local pain generated by a bee sting, PLA_2_ is the major allergen factor of the venom, alongside hyaluronidase and melittin [39,41,134,135,136]. Generally, after an accidental exposure to hvPLA_2_, the human body reacts by producing IgG1 anti-PLA_2_ antibodies with low affinity. Repeated contact with the HBV in beekeepers and venom-treated patients (see Section 4.8 and  Section 5) results in an increase in IgG4 anti-PLA_2_ antibodies with high affinity [137]. On the other hand, in very rare situations, IgE anti-PLA_2_ antibodies trigger allergy in HBV-sensitive patients [138]. These outcomes were also reported after in vitro tests using human leukocytes [136]. Additionally, PLA_2_ is responsible for IgE-mediated anaphylactic shock [45,139].

Taking into account the correlation between the whole content [47], and the PLA_2_ content [49] of the *A. mellifera* venom gland and the bee’s age, a differentiated immune response occurs after stinging by young or adult bees. Analysing IgG4 polyclonal antibodies from beekeepers (highly and most frequently exposed to the HBV), Schneider et al. [121] showed that from the hvPLA_2_ only specific epitopes (not the entire molecule) are responsible for its actual immunogenicity. Experimental data revealed that in the hvPLA_2_ molecule, there are four such epitopes. The first one is the region containing the glycosylated N^46^, and the other three are PLA73-96, PLA107-125 and PLA140-164 [140,141]. An important role in the modulation of the immune answer to this powerful allergen is played by the enzyme catalytic site. This involvement was proved by Annand et al. [63] who tested the response of basophils and the synthesis of IgG and IgE using both normal and mutant hvPLA_2_s. Changes in charge, amino acid sequence or conformation of hvPLA_2_ lead to variations in its allergenic activity [135]. Whether the glycosylation does or does not account for the immune response could be considered at least controversial, and more experimental and clinical data (on larger study groups) is required. Thus, it was shown that the sugar residues from the hvPLA_2_ were less important [142] or not involved at all in the allergenicity [121]. On the other hand, Prenner et al. [143] concluded that the hvPLA_2_ fucosilated N-acetylglucosamine residue could play an immunogenic role. Also, Dudler et al. [144] analysed the specificity of the human T cell response against hvPLA_2_ and identified some T cell clones activated by the entire enzyme, but not by its non-glycosylated variants. Additionally, the active hvPLA_2_ induced an IgE response in mice, while the mutant, inactive form of the enzyme did not [145]. Similar results were also reported by Förster et al. [8], on both natural hvPLA_2_ and recombinant PLA_2_ produced in *Escherichia coli.* In the same line, it has been shown that most individuals with HBV allergy (96%) also react to the recombinant PLA_2_ produced in *Escherichia coli* [146]. For detailed reviews see Perez-Riverol et al. [147] with roles of hvPLA_2_ (and from other species) in allergy, and Blaser et al. [148] for the immunology of hvPLA_2_ in allergic and non-allergic subjects.

### 4.6. Anticoagulant Effect

Once arrived in the blood flow, apart from its haemolytic activity, hvPLA_2_ also possesses another converging, anticoagulant effect [114,149]. Venom PLA_2_s have been classified by Boffa et al. [150] into 3 categories, based on their anticoagulant activities: strong, moderate (including hvPLA_2_) and weak. Comparing different phospholipases, Kini and Evans [151] pointed out a part of the molecule situated between residues 54 and 57, which is responsible for this effect. In strongly anticoagulant PLA_2_s, this region is positively charged and contains 4×K residues located on the surface of the molecule, while in the enzymes from the other categories, it is negatively charged [151]. Interestingly, while all strong anticoagulant enzymes are basic proteins, not all basic PLA_2_s have strong anticoagulant action [150,152]. Thus, the exact location of the basic residues is the determining factor, rather than its overall basicity [151]. In hvPLA_2_, the region thought responsible for its anticoagulant effect contains 3 positively charged residues and has a basic character that explains the moderate effect of the enzyme [151]. Condrea et al. [153,154] have shown that alterations of this region of the molecule can lead to a loss of its anticoagulant properties. Billy et al. [114] noted that, in activated platelets, cobra venom PLA_2_ inhibited the production of thrombin and the annexin-V binding; however, there was no observable effect on clotting or the activation of platelets. Mukherjee et al. [155], also working on cobra venom, proved its inhibitory potential on thrombin and factor Xa, non-enzymatically, and without relying on phospholipids. Kini [156] reviewed the various action mechanisms of anticoagulant snake venom PLA_2_s. Studying HBV, Ouyang et al. [157] also pointed out the same inhibition of the activation of prothrombin; however, they concluded that this effect could be attributed to the suppression of the procoagulant properties of intact phospholipids.

### 4.7. PLA_2_ and Apoptosis

hvPLA_2_ can be involved in the first steps of apoptosis, after a mechanism similar to that of the tumour necrosis factor (TNF) in some leukaemic cell lines. Melittin (as well as TNF) has the ability to activate the hvPLA_2_ and also the cytosolic PLA_2_ (from mammalian cells). Participation of PLA_2_ in apoptosis has not been fully elucidated. It is assumed that a crucial role is performed by the arachidonic acid release from membrane phospholipids. It is then metabolised (via lipooxygenase and cyclooxygenase) with the production of free radical oxygen species, involved in cytolysis. PLA_2_ is also responsible for ceramide production, which in turn has an important role in the TNF or the HIV-induced apoptosis [158]. Anyway, it was proved that prevention of PLA_2_ activation was followed by resistance of the studied cell lines to TNF [159,160].

Additional experimental data showed the relevance of cellular PLA_2_ (cPLA_2_) in apoptosis. Thus, the inhibition of PLA_2_ further inhibited DNA fragmentation [161]. Other in vitro studies showed increased activity of cPLA_2_ in HeLa and breast carcinoma cells consecutive to induction of apoptosis with TNFα [159,162] (observation consistent with the report of Wu et al. [160]), or in neurons exposed to oxidative stress and β-amyloid [163].

On the other hand, Kim et al. [164] showed that the hvPLA_2_ injected intraperitoneally (0.2 mg/kg) significantly alleviated the diethyl 1,4-dihydro-2,4,6-trimethylpyridine-3,5-dicarboxylate diet-induced apoptosis in mice, associated with an important reduction in the cleaved caspase-3 levels. Unfortunately, the paper did not explain the molecular mechanisms involved in this protection. The same authors further claimed that the hvPLA_2_ reduced hepatic fibrosis and demonstrated the anti-inflammatory effect by reducing the number of neutrophils, F4/80+ macrophages and CD4+ T-cells in the liver tissue in the same experimental conditions, but such results should be considered with some reserve since they incorrectly compared Portal regions with central regions of lobules. Another study demonstrated that hvPLA_2_ prevented in vitro the apoptosis of spleen regulatory T cells via a concomitant decrease in their caspase-3 expression and of annexin V-positive early apoptotic populations [165]. Also, the regulatory T cells treated with hvPLA_2_ showed a higher expression of programmed cell death-1 protein (that also helps cancer cells to elude the immune response [166]) and cytotoxic T-lymphocyte-associated protein-4 (an inhibitory receptor from the CD28 immunoglobulin subfamily (reviewed in [167])).

It is difficult to comment on the diverging data reported by different groups of researchers, but it is very likely that progress and higher availability of laboratory tools aimed to investigate the molecular mechanisms of the various cell death subroutines (as recommended by the Nomenclature Committee on Cell Death 2018—[168]) will eventually solve this problem too.

### 4.8. Antiviral, Antibiotic and Antitumoral Effects of hvPLA_2_

Given the presence of phospholipids in the viral envelope and in the membranes of pathogenic microorganisms and PLA_2_’s ability to hydrolyse them, numerous studies have been conducted, assessing the utility of the enzyme in fighting infections or cancer. Fenard et al. reported the successful use of hvPLA_2_ [169], or of a peptide (p3bv) derived from this enzyme [170] in preventing HIV entry into the cell and its further multiplication. However, according to these authors, the peptide p3bv proved to be less effective as compared to the entire molecule. The same mechanism was described by Santos et al. [171], studying the antiviral effect of *Crotalus durissus terrificus* PLA_2_ on the Chikungunya virus. hvPLA_2_ had a moderate virucidal effect on *Flaviviridae* (hepatitis C, dengue and Japanese encephalitis virus) at relatively low concentrations, that did not cause cytotoxicity or haemolysis [172]. Further studies on the same virus family showed a more potent virucidal activity of various snake venom PLA_2_s [172,173]. Muller et al. [174] noted that PLA_2_ only affects enveloped viruses, proving that its action mechanism depends on the presence of glycerophospholipids in the viral envelope. For a review of the antiviral effects of HBV and its components, including hvPLA_2_, see Yaacoub et al. [9].

On the other hand, hvPLA_2_ has an antimicrobial effect (but quite a reduced one), acting on the membranes of Gram-positive bacteria [105,116,175], at a comparable efficiency to the homologous enzyme isolated from cobra venom, but around 100 times less potent than sPLA_2_ of human cells [176]. In this case, the presence of Ca^2+^ was shown to be the modulating factor of enzyme activity [105]. Boutrin et al. [177] proved the bacteriostatic and bactericidal effect of hvPLA_2_ on Gram-negative bacteria, with minimum bactericidal concentrations in 2 h cultures being 10^−5^–10^−6^ mg/mL. Additionally, hvPLA_2_ even in very low concentrations had a lethal effect on *Trypanosoma brucei brucei*; this effect could be explained by direct membrane lysis or by interference with Ca^2+^ levels [177]. Furthermore, PLA_2_s have antimalarial properties [178,179]. Deregnaucourt and Schrével [178] showed that both toxic and non-toxic sPLA_2_s had a lethal effect on the intraerythrocytic development of *Plasmodium falciparum* via serum phospholipid hydrolysis; interestingly, hvPLA_2_ was stage-specific, only being active in the 19–26 h period of the 48 h cycle of the parasite. This effect is not linked to the actual binding of the enzyme to erythrocyte membranes, but rather to the presence of serum lipoproteins that are hydrolysed by PLA_2_ into arachidonic, linoleic, or docosahexaenoic acid (toxic extracellular compounds that induced the direct degeneration of *Plasmodium* without damaging the host cell membrane) [179]. Moreira et al. [180] showed that, through the expression of the hvPLA_2_ gene in transgenic mosquitoes, the development and transmission of the parasite were seriously impaired, leading to a possible way of controlling this disease.

Finally, PLA_2_s also have antitumor effects. The toxic and cytostatic effects of the hvPLA_2_ were demonstrated in vitro on various lines of tumoral cells, such as MCF-7 cells [181], or renal cancer cells [182]. hvPLA_2_ generated a moderate inhibition of their proliferation; however, its effect was much enhanced by the co-administration of phosphatidylinositol-(3,4)-bisphosphate [183]. Further studies using phosphatidylinositol homologues proved that the 3-phosphorylated ones had the strongest antitumoral properties when combined with hvPLA_2_; those with a polyunsaturated fatty acid as an acyl group had a reduced synergy with hvPLA_2_ [182]. Furthermore, since HBV displays a marked synergic effect of hvPLA_2_ and melittin [184], it can serve as an even more powerful antitumoral agent [185,186,187]. For reviews on the applications of HBV in cancer therapy see [10,12,188]. With an interesting approach, Shi et al. [189] reviewed the pharmacological effects and mechanisms of the HBV (including the hvPLA_2_) and its main components in inflammatory diseases, pain and neurological disorders, microbial diseases, cancer, and in liver, kidney, lung and muscle injury. Other PLA_2_s have also been observed to have similar properties: the enzyme obtained from *Bothrops jararacussu* venom exhibited a strong antitumoral and antimetastatic effect on triple-negative breast cancer cells, through numerous mechanisms, including induction of apoptosis and autophagy, decreasing cell proliferation and migration, as well as blocking the epithelial–mesenchymal transition [190]. Bazaa et al. [191] demonstrated that the *Macrovipera lebetina transmediterranea* venom PLA_2_ was a powerful inhibitor of both adhesion and migration of human tumour cells, an effect that was mediated by α5β1 and αv-containing integrins and did not rely on the integrity of the catalytic centre of the molecule. Furthermore, peptides derived from the C-terminal region of snake venom PLA_2_s, despite lacking catalytic activity, proved to be potent cytotoxic agents, with an efficiency comparable to that of paclitaxel in murine models of breast tumours [192].

### 4.9. Toxicity of hvPLA_2_

As the second most prevalent active molecule, hvPLA_2_ highly contributes to HBV toxicity. With low toxicity when pure, it is activated by high doses of melittin [193,194,195,196], the main component of the HBV, and turns into a major haemolytic factor [7,45,197]. This is an illustrative example of how natural selection facilitated the synthesis of two very different molecules (a protein and a peptide) by the same insect organism and with the same purpose.

hvPLA_2_ has a similar lytic activity to that of the PLA_2_ from cobra venom, more powerful as compared to the enzyme isolated from the viper venom [7], and approximately 100 times more potent than that of human sPLA_2_s [176]. Within the genus *Apis*, the venom of *A. mellifera* (tested from three populations, *A. dorsata*, *A. cerana*, *A. florea*) demonstrated similar lethal activity toward mice [33]. When comparing the toxicity of European and Africanized HBV, the LD_50_s were also similar [50,198]. Therefore, it remains possible that the activity of hvPLA_2_ from all these bee species could be similar. While Ownby et al. [195] used the hvPLA_2_ in a dose of 4 mg/kg with intramuscular administration in mice, we calculated and tested on rats an LD_50_ of 9.3 mg PLA_2_/kg via subcutaneous route [13,14]. Other researchers reported in mice detailed effects of snake venom PLA_2_ from Indian cobra (*Naja naja naja*) venom (LD_50_ of 2.4 mg/kg) [199] or crossed pit viper (*Bothrops alternatus*) venom (LD_50_ of 140 µg/kg) [200], in both cases administered by intraperitoneal route. The differences in the tested doses also confirm the different degrees of lethality of the enzyme from different genera or species.

PLA_2_ toxicity was first explained by its enzymatic effect on the membrane phospholipids. Thus, the membrane integrity is compromised, with consequences on the functions of the targeted cells. Moreover, the fatty acids and the lysophospholipids produced in high amounts by the enzymatic digestion propagate the membrane damages by detergent-like effects; the arachidonic acid triggers an accentuated oxidative stress followed by increased lipid peroxidation and by oxidative damage to membrane proteins [124,201]. All the structural alterations of the membranes affect their permeability, mainly for Ca^2+^ ions, whose cytosolic increase could trigger cytoskeletal changes, lipolysis and proteolysis [124].

**Figure 5:**
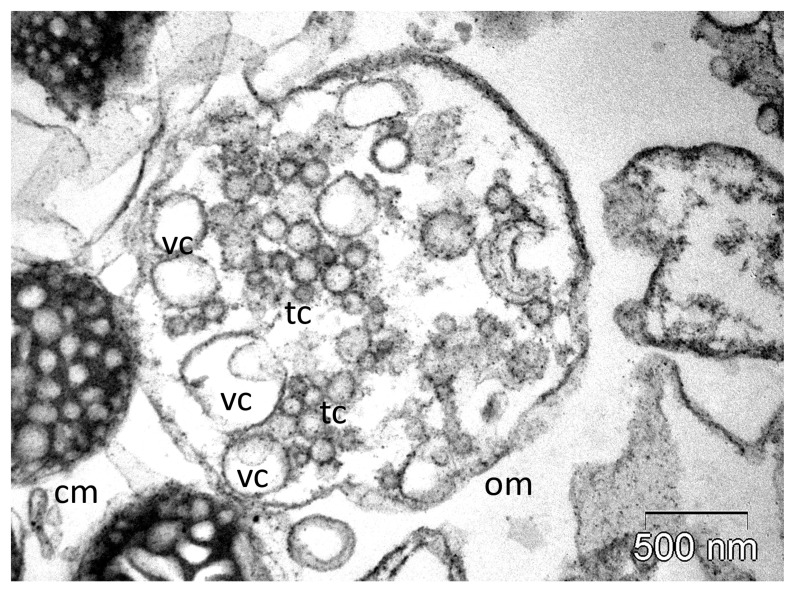
Severe ultrastructural alterations of orthodox mitochondria isolated from rat adrenocortical cells, generated in vitro by experimental administration of a high dose of hvPLA_2_ (personal archive A.F.). cm, condensed mitochondrion; om, orthodox mitochondrion; tc, tubular cristae; vc, vesicular cristae (abnormal) (personal archive A.F.).

As a cytolytic factor, PLA_2_ is involved in mast cell lysis, and therefore, in histamine [202] and serotonin [38] release. Furthermore, it causes K^+^ release from skeletal muscle cells, and epinephrine release from adrenal medulla [38]. PLA_2_ activation also results in the inhibition of oxidative phosphorylation. It attacks the succinate-dehydrogenase (and other enzymes catalysing metabolic dehydrogenation), as well as the respiratory chain-containing membrane. We previously reported the ability of the hvPLA_2_ to induce dose-dependent fusions of the adrenocortical mitochondria cristae in vitro, resulting in large vesicular cristae (some with 2–3 membranes, as can be observed in Figure 5), that further collapsed at higher doses [15]. Similar but less pronounced ultrastructural alterations of the adrenocortical mitochondrial cristae (along with other cellular responses) were found when we tested in vivo the subcutaneous administration of hvPLA_2_ (in a dose of 9.3 mg PLA_2_/kg) [14]. PLA_2_ inhibits the tissular thromboplastin and destroys the lysosomes. Moreover, the free fatty acids released during the phospholipid breakdown interfere as well with oxidative phosphorylation; they also produce changes in membrane permeability by different mechanisms [130,201]. All these effects may participate in tissue damage resulting after the local application of venom [7,38].

PLA_2_ displays a marked neurotoxicity. In contrast to other enzymes (for example PLA_2_ isolated from viper venoms—*Vipera ammodytes meridionalis* or *Vipera russelli formosensis*), which act in combination with other molecules [203], hvPLA_2_ exerts its neurotoxic action mostly alone. On the brain cell membranes, there are located receptors to which the hvPLA_2_ binds with high affinity [204,205]. These receptors, called N-type receptors, are believed to represent binding targets for endogenous sPLA_2_s, but they also have an important role in the progress of the molecular phenomena involved in PLA_2_ neurotoxicity.

Continuing their studies in this direction, the same research group reported interesting in vivo responses of neurons to the hvPLA_2_, via N-type receptors [205,206].

Experiments with mutated PLA_2_ (with different sequences of amino acids at different levels of the enzyme) showed that the hvPLA_2_ high affinity for the N-type receptors is essentially provided by the amino acids located in the interfacial binding domain (amino acids 109–124), in the Ca^2+^ binding region, and at the N-terminal end, respectively [123]. On the other hand, the same authors noted that mutant hvPLA_2_ molecules (with low affinity for these receptors) are devoid of neurotoxicity even though some of them still maintain a high enzymatic activity [123].

PLA_2_ neurotoxicity also results, as discussed above, from the direct action of the enzyme on the phospholipids from the membranes of the various structures participating in the blood–brain barrier and from neuronal membranes, respectively. All the damage and cascade events are eventually responsible for the structural and functional modifications of neurons. We found important ultrastructural alterations of the frontal cortex neurons consecutive to experimental testing of hvPLA_2_ (unpublished results), facilitated by a compromised blood–brain barrier. Some of these changes were consistent with those (histological, histochemical, ultrastructural and physiological) reported by us consecutive to the experimental administration of HBV [207]. Apart from affecting the central nervous system, the HBV molecules play an important role at the level of the peripheral nervous system as well. In the same paper, we also mentioned the immediate reactions of rats to the high dose of HBV including increased aggressiveness, motor agitation and uncoordinated movements, abnormal discharges (at 40–45 min after the HBV injections), piloerection, lethargy and generalised convulsions crisis (after 1.5 h) [207]. Such symptoms were previously described consecutive to the intraperitoneal administration of hvPLA_2_ in mice (at an LD_50_ of 2.4 mg/kg) [199], or when hvPLA_2_ was injected in rats via the intracerebroventricular route [208]. Pungerčar and Križaj [209] attributed many of these toxic effects of the hvPLA_2_ to its action on the membranes of motoneurons participating in the neuromuscular junctions. The hvPLA_2_ neurotoxicity was further demonstrated in vitro. Thus, it was shown that, once it arrived in synaptosomes, hvPLA_2_ caused the uncoupling of mitochondria [210], with a decrease in the ATP synthesis, and subsequent increase in cytosolic Ca^2+^ ions that modulated the frequency of neurotransmitter release.

In snake venom envenomation, the cancelling of neuromuscular transmission results in respiratory failure, paralysis and eventually death, PLA_2_ blocking both cholinergic synapses (in skeletal muscles), and non-cholinergic neurons (from the cerebral cortex, hippocampus and granular layer of the cerebellum [209]).

Another mechanism explaining the cellular toxicity of hvPLA_2_ is based on its property to bind to calmodulin (cytosolic receptor for Ca^2+^) [211] and, this way, to modulate many metabolic pathways. However, this mechanism requires more investigations.

On the other hand, the arachidonic acid released during the enzymatic action of PLA_2_ on membranes stimulates glycogenolysis in cortical astrocytes [212], in order to energetically sustain the resistance of glial and neuronal cells against the deleterious effects of PLA_2_. Katsuki and Okuda [213] showed that the arachidonic acid also contributes to neurodegeneration. Farooqui et al. [130] reviewed more molecular mechanisms involved in PLA_2_ neurotoxicity.

However, there is contrasting data revealing a potential neuroprotective effect of hvPLA_2_ in a mouse model of Alzheimer’s disease, induced by lipopolysaccharide injection [214]. Injected intraperitoneally, the enzyme was shown to reduce the memory impairment and amyloidogenesis, through the inhibition of nuclear factor kappa B, glial fibrillary acidic protein and allograft inflammatory factor 1 expression, as well as through reduced cytokine release. Additionally, hvPLA_2_ increased the number of regulatory T cells, which in turn led to a reduction in microglial activation. Converging results were also reported by the same authors in vitro, using BV-2 cells.

hvPLA_2_ is also a myotoxic compound. Apart from the N-type receptors located on the neuronal plasma membranes, experimental data proved a potent expression of M-type receptors for sPLA_2_s in muscular tissues (skeletal and smooth muscles, myocardium). In addition to this, the presence of certain plasma PLA_2_-binding proteins suggests that the bee enzyme is involved in vivo in a series of other still unknown physiological processes [63,68,123].

Studies on mice showed myotoxic effects of the hvPLA_2_ injected intramuscularly, consisting in extensive ultrastructural alterations that culminated with cellular necrosis [195], or degenerated muscle fibres and necrosis around the injection site, causing respiratory distress and paralysis [199]. Gutiérrez and Ownby [215] explained in detail the mechanisms of PLA_2_s (including the hvPLA_2_) myotoxicity, emphasising the differences between the local and systemic effects.

Working with snake and mammal pancreatic PLA_2_s, Lambeau et al. [216] revealed that G^30^, and D^49^ as well as L^31^ are essential for PLA_2_ binding to the M-type receptors. Furthermore, Lambeau et al. [204] showed that the receptors for sPLA_2_s differ in different tissues, being responsible for “tissue-dependent pharmacological profiles” and that in the skeletal muscles, their density is age-dependent.

**Figure 6:**
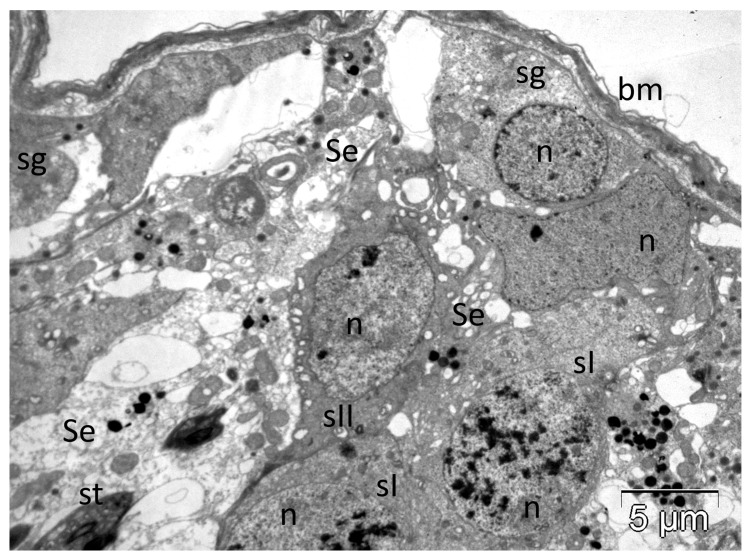
Severe ultrastructural alterations of Sertoli cells in a seminiferous tubule, generated in vivo by experimental administration of a high dose of hvPLA_2_ in rats. bm, basement membrane; n, nucleus; sI, primary spermatocyte; sII, secondary spermatocyte; Se, Sertoli cell; sg, spermatogonia. (personal archive A.F.).

hvPLA_2_ triggers toxic effects in other organs too. The M-type receptors for sPLA_2_s were found not only on the membranes of muscle cells but also in kidney, lung, liver and pancreas [63,68,123,204], with roles that remain to be elucidated. We found important ultrastructural changes produced by the experimental administration of the hvPLA_2_ in several organs of rats. To date, we have demonstrated the ability of the hvPLA_2_ to damage the interstitial Leydig cells and to cancel the permeability of the testicular barrier, thus interfering with the normal progression of spermatogenesis. The latter effect was facilitated via extensive ultrastructural damage of the Sertoli cells [13], as shown in Figure 6. In the adrenocortical cells, apart from the above-mentioned changes in mitochondria, the hvPLA_2_ also triggered dose-dependent alterations of nuclei and smooth endoplasmic reticulum, associated in some cases with plasma membrane rupture and the release of organelles outside the cells [14].

However, low doses of hvPLA_2_ (0.2 mg/kg injected intraperitoneally) seem to have a hepatoprotective effect against the acetaminophen (500 mg/kg)-induced acute liver injury [217] in mice. To demonstrate this effect, the authors also tested and showed reduced protection of hvPLA_2_ in regulatory T cells-depleted mice, establishing a correlation between this cellular population and the normal functional parameters of the liver.

As a main constituent of HBV, hvPLA_2_ also highly contributes to the overall effectiveness of the venom consecutive to massive attacks of both European and Africanized honeybees on humans [3,218,219,220,221], or animals (dogs [222,223], or horses [224,225]).

The toxic effects debuted in humans with local reactions and culminated with extensive haemolysis [7], circulatory and respiratory impairment, acute renal failure (after 200–500 stings in adults) and death, after more than 500 stings (Refs. [4,218,219,226], and reviewed by Schmidt [227]).

## 5. Immunotherapy

As mentioned above, hvPLA_2_ is a powerful allergen. Thus, one of its main medical applications is in immunotherapy. Early studies showed that doses of 100 μg of HBV, administered constantly, over a period of several years, provided protection against allergic reactions [52] and induced the production of IgG, while decreasing IgE levels [228,229]. Patient immunisation using only hvPLA_2_ was proved equally efficient in conferring the same protective effect against bee stings [230]. Müller et al. [139] tested 3 peptides derived from hvPLA_2_ that contained its epitopes and noted a similar success of the immunotherapy as that obtained using whole HBV. Furthermore, their results showed a decrease in the frequency of allergic side effects when using the peptides, since they lacked catalytic activity [139]. Additionally, Nair et al. [231] proved that there was no cross-protection given by IgG antibodies against hvPLA_2_. However, antibodies against bald-face hornet venom offered protection against hvPLA_2_, as well as PLA_2_s from yellow hornet or yellow jacket.

In an interesting approach, Jilek et al. [232] assessed the effectiveness of genic therapy in vivo, on mice injected with plasmids carrying a PLA_2_ gene sequence. They reported long-time low levels of IgE and IgG1 (and increased levels of IgG2a and IgG3) consecutive to the administration of the recombinant plasmids, both before and after the mice sensitization to hvPLA_2_. They also demonstrated that the prophylactic treatment was more effective than the therapeutic one, this way opening the possibility of using such DNA vaccines for human immunotherapy.

## 6. Anti-PLA_2_ Molecules

Due to the numerous adverse effects of PLA_2_s, the inhibitory action of various substances has been extensively studied. These molecules act on different, specific, targets to inactivate the enzyme. Several natural compounds with such properties have been identified, including manoalide, luffariellolide, and scalaradial, isolated from marine organisms [233]. The latter was proved to have a two-step action mechanism, with an initial non-covalent attachment, followed by a covalent modification of hvPLA_2_ near its substrate-binding site [234], namely of K^127^ [104]. In 1995, De Rosa et al. [235] discovered and isolated from a species of marine sponge, *Fasciospongia cavernosa*, a compound (cacospongionolide B) with a strong anti-inflammatory activity mainly given by its capacity to inhibit PLA_2_. Because of its importance, not only as a possible anti-venom agent but also with applications in different inflammatory affections such as asthma, psoriasis or rheumatoid arthritis, analogues of this natural product were synthesised, that preserve the inhibitory activity on the hvPLA_2_ [236]. Antimicrobial peptides, such as magainin 2, indolicidin, and temporins B and L, all derived from natural sources, were also observed to lower the catalytic activity of hvPLA_2_ [237]. Furthermore, numerous other proteins (lipocortin, bovine serum albumin, myoglobin, lysozyme) showed an inhibitory effect, but only in experiments using low substrate liposomes [238]. Hains et al. [239] purified another PLA_2_ inhibitor from the serum of the Australian tiger snake (*Notechis ater*), composed of two protein subunits that form a complex of approximately 110 kDa; upon further experiments, this molecule was observed to block all the different PLA_2_ enzymes tested. A similar behaviour was noted in proteins from the blood of different cobra species [240,241]. On the other hand, another molecule isolated from *Crotalus durissus terrificus* serum was proved to have a specific action only against the snake PLA_2_ neurotoxin (by binding to its active site) and not against hvPLA_2_ or other PLA_2_s [242,243,244]. In the serum of *Agkistrodon blomhoffii siniticus*, three proteins were described, the first two with inhibitory effects on PLA_2_s from *Crotalidae* venom (either acidic or basic), and another one that blocked all PLA_2_s tested, including hvPLA_2_ [245]. Noetzel et al. [203] also reported a series of substances, including vitamin E, with anti-PLA_2_ effect on the enzyme secreted by other species. Apart from the exogenous PLA_2_ inhibitors, it has been observed that crotapotin, a peptide that is naturally complexed to the *Crotalus durissus terrificus* venom PLA_2_, acts as an intrinsic enzymatic inhibitor; its action, however, is not restricted to the sPLA_2_ of the same species, as studies have noted its potency on hvPLA_2_ and on other snake PLA_2_s [246]. Many arthropod venoms, including HBV, as well as snake venoms, contain citrate that acts as an endogenous inhibitor of the enzyme, mainly through its ability to bind Ca^2+^ [247,248]. Synthetic calcium chelators, such as EDTA, can also reduce the activity of Ca^2+^-dependent PLA_2_s [249]. Indoxan was proved to be effective in blocking PLA_2_-induced cell damage, through reduced generation of arachidonic acid and prostaglandin D2 [250]. A similar mechanism was also reported for nordihydroguaiaretic acid and aristolochic acid [251]. Heparin is another molecule with a powerful, non-specific, inhibitory action [246,252]. Furthermore, numerous other chemical substances with the same property, such as benzenesulfonamides [253] or 4-bromophenacyl bromide [195,254] have been described in the literature. The role of these inhibitors is paramount, since through their development the roles of different PLA_2_ classes could be determined [255,256,257]. Crous et al. [258] reviewed the interactions of the human and snake sPLA2s with several inhibitors using X-ray crystal structures and discussed potential therapeutic applications of these inhibitors.

## 7. Concluding Remarks

In conclusion, PLA_2_ is one of the main toxic components of the HBV, commonly found in many other animal species as well, being partially responsible for the venom activity. It is a molecule of high interest for the scientific community (including our department) due to its diverse effects. Far from simply hydrolysing phospholipids, this enzyme plays numerous important biological roles. It interacts non-specifically or specifically with membranes, by mechanisms discussed in detail, being both a powerful toxin and an immune response modulator. The diverse effects of the hvPLA_2_ recommend it as a promising candidate for various medical applications. This molecule also represents a powerful tool in basic research, useful for deepening our understanding of the protein-lipid and protein–protein interactions in biological membranes. The importance of PLA_2_ is reflected by the number of studies focused on finding inhibitors for counteracting its toxicity. Analysing the literature data, we noted that some aspects (the structure and the molecular mechanism) were more intensively studied than others. Therefore, more attention should be paid to the clinically relevant ones such as the anticoagulant effects of the hvPLA_2_ (important for patients predisposed to thrombotic events), immunotherapy or apoptosis. For future research perspectives, we plan to deepen the understanding of hvPLA_2_ interaction with cellular structures in vivo by revealing ultrastructural responses of other tissues.

## Data Availability

No new data were created or analyzed in this study.

## Abbreviations

The following abbreviations are used in this manuscript:

A.m.	*Apis mellifera*
HBV	honeybee venom
hv PLA_2_	honeybee venom phospholipase A_2_
HPLC	high performance liquid chromatography
PLA_2_	phospholipase A_2_
TNF	tumour necrosis factor
